# A curve shortening flow rule for closed embedded plane curves with a prescribed rate of change in enclosed area

**DOI:** 10.1098/rspa.2015.0629

**Published:** 2016-01

**Authors:** Michael C. Dallaston, Scott W. McCue

**Affiliations:** 1 Department of Chemical Engineering, Imperial College London, London SW7 2AZ, UK; 2 School of Mathematical Sciences, Queensland University of Technology, Brisbane, Queensland 4000, Australia

**Keywords:** curve shortening flow, geometric partial differential equation, extinction behaviour, pinch-off, coalescence, self-similar solutions

## Abstract

Motivated by a problem from fluid mechanics, we consider a generalization of the standard curve shortening flow problem for a closed embedded plane curve such that the area enclosed by the curve is forced to decrease at a prescribed rate. Using formal asymptotic and numerical techniques, we derive possible extinction shapes as the curve contracts to a point, dependent on the rate of decreasing area; we find there is a wider class of extinction shapes than for standard curve shortening, for which initially simple closed curves are always asymptotically circular. We also provide numerical evidence that self-intersection is possible for non-convex initial conditions, distinguishing between pinch-off and coalescence of the curve interior.

## 1. Introduction

This paper concerns the evolution of a plane embedded curve $\gamma :[0,T)\to R^{2}$, where the curve *γ*(*t*) at time *t* is represented by the position vector ***x***(*u*,*t*), and *γ* evolves with a velocity at *t* that is dependent on the shape of *γ*(*t*). Here the parameter *u* is an arbitrary parametrization. The most widely studied of such flows is *curve-shortening flow*, whereby the normal velocity of the boundary at a point is equal to the curvature at that point:

1.1 $$\frac{\partial \text{x}}{\partial t}\cdot \text{n}=k(u,t),$$

where ***n*** denotes an inward unit normal vector. The tangent velocity is specified by the choice of parameter *u* and has no effect on the evolution of *γ*. For this flow, it is known that embeddedness is preserved and that any initially simple closed curve will become convex in finite time [1], then shrink to a point, becoming asymptotically circular as it does so [2–4] (for a summary of this problem, see [5]). We refer to the shrinking of a curve to a point at t=T<∞ as finite-time extinction.

Here we are concerned with the generalization of (1.1):

1.2 $$\frac{\partial \text{x}}{\partial t}\cdot \text{n}=k(u,t)-q(t),$$

where *q* is determined by the specified constant change in area −d*A*/d*t*=*β*, as follows. Given *γ* is a family of embedded curves, smooth in space and time, the following identities hold [6]:

1.3 $$\int_{\gamma }k\;ds=2\pi ,\;\int_{\gamma }ds=L(t),\;\frac{dA}{dt}=-\int_{\gamma }\frac{\partial \text{x}}{\partial t}\cdot \text{n}\;ds,\;\frac{dL}{dt}=\int_{\gamma }k\frac{\partial \text{x}}{\partial t}\cdot \text{n}\;ds,$$

here *s* is the arc length parameter, and *L*(*t*) and *A*(*t*) are the length and area of *γ*(*t*), respectively. Integration of (1.2) around *γ* yields

$$-\frac{dA}{dt}=2\pi -q(t)L(t)$$

and rearranging for *q* results in

1.4 $$q(t)=\frac{2\pi -\beta }{L(t)}.$$

For the special case *β*=2*π*, then *q*=0, and thus (1.2) reduces to standard curve shortening flow (1.1). For *β*≠2*π*, our flow rule (1.2), (1.4) is non-local, as it depends on *L*(*t*). The case *β*=0 corresponds to an *area-preserving flow*, which has been studied previously [7–10].

The flow rule (1.2), (1.4) arises in the study of contracting bubbles in a fluid mechanics problem consisting of viscous fluid confined between two parallel plates (a Hele-Shaw cell), in which a velocity and curvature term appear in the free boundary (dynamic) condition [11] (the equivalent rule is presented in eqns (27)–(28) in [11], although both space and time are scaled differently there). The velocity term in the Hele-Shaw problem models the effects of kinetic undercooling [12,13]. An equivalent model arises from an ideal Stefan (phase change) problem which includes a Gibbs–Thomson condition on the solid-melt interface with an additional kinetic term [6,14–16]. While the full problem in [11] consists of a field equation (Laplace's equation) exterior to the bubble, in the limit that the bubble boundary is small, the only contribution from the exterior problem is to force the area inside *γ* to decrease at a prescribed rate *β*. Importantly, for the Hele-Shaw problem treated in [11], there is surface tension acting on the bubble boundary, with a surface tension parameter *σ* which is related to *β* via *σ*=2*π*/*β*. Thus, *σ*=1 corresponds to standard curve shortening flow. Physically, we expect higher surface tension (*σ*>1 or 0<*β*<2*π*) to make evolving curves even more round, thus intuition suggests that the key results for closed convex curves in standard curve shortening flow to carry over to (1.2), (1.4) for 0<*β*<2*π*. At the other end of the parameter space, a very large value of *β* corresponds to weak surface tension. The extreme case *σ*=0 (or β=∞) is not considered here; after a rescaling in time, it leads to

$$\frac{\partial \text{x}}{\partial t}\cdot \text{n}=1,$$

which does not involve smoothing via curvature so that convex curves can develop corner singularities in finite time before extinction (at *t*=1/*sup*{*k*(*s*,0)}) [17,18].

More generally, our flow rule (1.2), (1.4) is one of a number of non-local generalizations of standard curve shortening flow (1.1). Apart from the area-preserving flow rule already mentioned, other relevant generalizations include a signed-area-preserving flow [19], a length-preserving flow [20,21] and the gradient flow of the isoperimetric ratio *L*^2^/4*πA* [22]. Non-local flows like (1.2) with *k* replaced by 1/*k* have also received attention recently [23–26]. While we will not be treating flows of hypersurfaces in higher dimensions [27,28], it is worth noting that non-local generalizations of mean curvature flow are also of interest [9,29,30]. A local variation of (1.2) is considered in [6], in which anisotropy is introduced by multiplying the velocity and curvature terms by angle-dependent coefficients. In that study, however, the term equivalent to *q* in (1.2) is taken to be constant in time, and the specifics of extinction behaviour and self-intersection, which we address in the current paper, are left as open problems.

Although the results contained in this paper rely on short-time existence of solutions to (1.2), which we do not address in this paper, the close relationship between (1.2) and area-preserving flow suggests that a proof of short time existence will follow in the same way as discussed in [4,8].

In this study of (1.2), (1.4), we are concerned with area-decreasing flows, *β*>0. Following Gage [8], the Cauchy–Schwartz inequality implies that

$$\int_{\gamma }k^{2}\;ds\geq \frac{4\pi^{2}}{L},$$

so the rate of change in length is bounded:

1.5 $$\frac{dL}{dt}=-\int_{\gamma }k^{2}\;ds+\frac{2\pi (2\pi -\beta )}{L}\leq -\frac{2\pi \beta }{L}.$$

Thus, for *β*>0, the length is certainly decreasing and so (1.2), (1.4) can be considered a generalized curve shortening flow rule. Furthermore, (1.5) implies that

$$\frac{d}{dt}(L^{2}-4\pi A)\leq 0,$$

which suggests that finite-time extinction is possible (as the isoperimetric inequality *L*^2^≥4*πA* forces the isoperimetric difference in brackets to be non-negative and we must have both limits L→0 and A→0 at extinction).

A further straightforward result for simple closed convex curves is found by using Gage's inequality [2]

1.6 $$\int_{\gamma }k^{2}\;ds\geq \frac{\pi L}{A}.$$

**Lemma 1.1**

An initially convex curve evolving according to (1.2), (1.4) with 0<β<2π will approach a circle in the limit it shrinks to a point.

**Proof.**

Using (1.6), which holds for simple closed convex curves, and following Gage [8], we find by direct calculation that

$$\frac{dL}{dt}=-\int_{\gamma }k^{2}\;ds+\frac{2\pi (2\pi -\beta )}{L}\leq -\frac{\pi }{L}\left(\frac{L^{2}}{A}-4\pi +2\beta \right),$$

which implies

$$\frac{d}{dt}\left(\frac{L^{2}}{A}-4\pi \right)=\frac{2L}{A}\frac{dL}{dt}+\frac{L^{2}}{A^{2}}\beta \leq -\frac{\left(2\pi -\beta \right)}{A}\left(\frac{L^{2}}{A}-4\pi \right),$$

This result, together with Bonnesen's inequality, gives

1.7 $$\frac{\pi^{2}}{A}{\left(r_{\mathrm{out}}-r_{\mathrm{in}}\right)}^{2}\leq \frac{L^{2}}{A}-4\pi \leq CA^{2\pi /\beta -1},$$

where *C*>0 is a constant, *r*_*out*_ is the radius of the smallest possible circle that encloses *γ*, while *r*_*in*_ is the radius of the largest possible circle contained within *γ*. Here *A* simply decreases like *A*=*β*(*T*−*t*), where *T* is the extinction time. Thus, at least for 0<*β*<2*π*, convex curves become more circular in shape as the area decreases, as we expect from our physical intuition mentioned above (this argument only provides convergence to a circle in the weak *C*^0^ sense). ▪

Another interpretation of (1.2), (1.4) is as a gradient flow. For a general flow rule, (1.3) leads to the result

$$\frac{d}{dt}(L^{2}-2(2\pi -\beta )A)=-\int_{\gamma }\frac{\partial \text{x}}{\partial t}\cdot \left[2L\left(k-\frac{2\pi -\beta }{L}\right)\text{n}\right]ds,$$

which implies that

$$\frac{\partial \text{x}}{\partial t}\cdot \text{n}=2L\left(k-\frac{2\pi -\beta }{L}\right)$$

is the steepest descent gradient flow of *L*^2^−2(2*π*−*β*)*A*. Thus, we see that (1.2), (1.4) can be thought of as the gradient flow of *L*^2^−2(2*π*−*β*)*A* after a simple rescaling in time, where the rescaled time $\tilde{t}$ satisfies $dt/d\tilde{t}=2L$ [23]. In this way, we again see that (1.2), (1.4) is a generalization of both standard curve shortening flow (1.1) (which is the gradient flow of *L*^2^ after the same time rescaling) and area-preserving flow (which is the gradient flow of the isoperimetric difference *L*^2^−4*πA* after the rescaling).

In this study, we are interested in the generalized curved shortening flow (1.2), (1.4) for the full range of possible values of *β*>0. Our goal is to explore the questions of whether finite-time extinction occurs and, if so, what shape does the curve evolve to in the extinction limit. These questions provide additional challenges when compared with the standard curve shortening rule (1.1) for two reasons:

(i) the extinction behaviour is much more complicated (due to the unboundedness of *q* in (1.4) as L→0);
(ii) non-convex curves will not always become convex without self-intersecting.

The paper consists of formal asymptotic and numerical results presented to investigate and suggest theoretical results regarding these two issues. In §2, we provide the theoretical results for an initially convex curve, showing that such a curve will remain convex. We then explore the range of possible extinction shapes using asymptotic and numerical techniques; these include not just circles, but also slits, and *n*-fold symmetric shapes which we only compute numerically. In §3, we demonstrate the possibility of two types of self-intersection. The first, which we refer to as ‘pinch-off’ is where the interior of *γ* becomes disconnected (the usage of the term ‘pinch-off’ is motivated by our application in fluid mechanics, for which a disconnected bubble interior is normally associated with the bubble pinching off and breaking up into two bubbles; in the present context, however, pinch-off does not imply there is a singularity in curvature). The second, which we call ‘coalescence’, is where the interior becomes doubly connected. We formulate conjectures on both types of self-intersection, namely that: coalescence is possible for 0<*β*<2*π* (as it is for the area-preserving flow in [10]) but not possible for *β*>2*π*; and pinch-off is possible for *β*>2*π* but not possible for 0<*β*<2*π*. We hope our theoretical, asymptotic and numerical results will stimulate further research to explore our conjectures using more rigorous techniques.

## 2. Extinction of convex shapes

### (a) Convexity is preserved

Unlike standard curve shortening flow (1.1), there is no guarantee that a curve evolving according to (1.2), (1.4) will remain simply connected in a given time interval. In §3, we show numerically that this is generally not the case. However, it is readily shown that an initially convex curve *γ*(0) remains convex under the flow rule (1.2), (1.4). As in [4], if *γ*(0) is strictly convex, the flow rule (1.2) may be written as a parabolic partial differential equation (PDE) for the curvature *k* as a function of time *t* and tangent angle *θ* from a reference direction. Note that convexity implies that *θ* is monotonic in arc length and thus may serve as a parametrization of *γ*(0).

The form of this PDE for a general flow rule is derived in [6,31], for example. We summarize the derivation here. Let ***x***=***x***(*s*,*t*) be parametrized by arc length *s* and consider a general normal velocity *V* (*s*,*t*). The evolution equation is

2.1 $$\frac{\partial \text{x}}{\partial t}=V\text{n}+v\text{t},$$

where ***t*** is the tangent vector and *v*=*v*(*s*,*t*) is the (as yet undetermined) tangent velocity for the arc length parametrization. Differentiating (2.1) with respect to *s* and taking normal and tangential components, we find

2.2 $$\frac{\partial V}{\partial s}+kv=\frac{\partial \theta }{\partial t},\;\frac{\partial v}{\partial s}-kV=0.$$

Here we have used the fundamental identities from differential geometry

$$\frac{\partial \text{t}}{\partial s}=k\text{n},\;\frac{\partial \text{n}}{\partial s}=-k\text{t},\;\frac{\partial \text{t}}{\partial t}=\frac{\partial \theta }{\partial t}\text{n},\;k=\frac{\partial \theta }{\partial s}.$$

The latter of (2.2) determines *v* uniquely up to a constant. The curvature evolves according to

2.3 $${\left.\frac{\partial k}{\partial t}\right|}_{s}=\frac{\partial^{2}\theta }{\partial s\partial t}=\frac{\partial^{2}V}{\partial s^{2}}+k^{2}V+v\frac{\partial k}{\partial s}.$$

Now we change parametrization to *θ*. The time derivative of *k* holding *θ* constant is given by the chain rule:

$$\begin{array}{rl} {\left.\frac{\partial k}{\partial t}\right|}_{\theta }={\left.\frac{\partial k}{\partial t}\right|}_{s}-\frac{\partial k}{\partial \theta }{\left.\frac{\partial \theta }{\partial t}\right|}_{s} & =k\frac{\partial }{\partial \theta }\left(k\frac{\partial k}{\partial \theta }\right)+k^{2}V+vk\frac{\partial k}{\partial \theta }-\frac{\partial k}{\partial \theta }{\left.\frac{\partial \theta }{\partial t}\right|}_{s}\\ & =k^{2}\left(\frac{\partial V}{\partial \theta^{2}}+V\right)-\frac{\partial k}{\partial \theta }\left[\frac{\partial \theta }{\partial t}-k\frac{\partial V}{\partial \theta }-vk\right]. \end{array}$$

The quantity in square brackets is identically zero, due to the first of (2.2). Thus, when *γ*(*t*) is parametrized by *θ* the curvature evolves according to

2.4 $$\frac{\partial k}{\partial t}=k^{2}\left(\frac{\partial^{2}V}{\partial \theta^{2}}+V\right).$$

This was the result derived in [6,31].

For the generalized curve shortening flow (1.2) considered in this paper, (2.4) becomes

2.5 $$\frac{\partial k}{\partial t}=k^{2}\frac{\partial^{2}k}{\partial \theta^{2}}+k^{2}(k-q(t)),\;\theta \in [0,2\pi ].$$

The solution to (2.5) represents a convex curve as long as *k* remains positive. Gage & Hamilton [4] showed that when *q*=0, the minimum *k* was non-decreasing, and the same argument holds for (2.5) when *q*<0 (as will become clear in the following proof). The same will not necessarily be true for *q*>0, but a similar argument may be made to provide a lower, positive bound, assuming *q* is bounded above. While *q* is not bounded as $t\to T^{-}$, we can establish that *γ*(*t*) cannot lose convexity at time *t*_1_<*T* strictly before the extinction time.

Let $k_{\min}(t)=\underset{\theta }{\inf}\{k(\theta ,t)\}$ and say *q*(*t*)<*Q* on an interval [0,*t*_1_]. Let *K*(*t*) satisfy

2.6 $$\frac{dK}{dt}=-K^{2}Q,\;K(0)=\frac{k_{\min}(0)}{2}\;\Rightarrow \;K=\frac{k_{\min}(0)}{2+Qk_{\min}(0)t}>0.$$

**Lemma 2.1**

*If q(t)<Q is bounded above in an interval [0,t*_1_*], the minimum curvature k*_*min*_*(t)≥K(t)>0 for t∈[0,t*_1_*].*

**Proof.**

Firstly note that $k_{\min}(0)>K(0)$ by construction. Suppose there exists a subsequent time when $k_{\min}(t)<K(t)$. Owing to the continuity of *k* there must be a time *t*_0_∈[0,*t*_1_], an *ϵ*∈(0,*K*(*t*_0_)), and point *θ*_0_ where

2.7 $$k(\theta_{0},t_{0})=k_{\min}(t_{0})=\epsilon ,\;\frac{\partial }{\partial t}(k-K)\leq 0,\;\frac{\partial^{2}k}{\partial \theta^{2}}\geq 0.$$

But from (2.5) and (2.6), we have

2.8 $$\frac{\partial }{\partial t}(k-K)=k^{2}\frac{\partial^{2}k}{\partial \theta^{2}}+k^{3}+K^{2}Q-k^{2}q.$$

As 0<*k*(*θ*_0_,*t*_0_)<*K* and *q*<*Q*, the right-hand side must be positive, leading to the contradiction. ▪

Note that from the above proof, the result that *k* is non-decreasing for *q*<0 clearly follows from setting *Q*=0, and does not require *q* to be bounded from below.

### (b) Formal results on extinction behaviour

#### (i) Definition of extinction shape

In our previous paper [11], we established some formal and numerical results on extinction shapes. Here we expand on these results. For definiteness, we define the concept of the *extinction shape* by rescaling. Suppose a curve *γ*(*t*) contracts to a point at ***x***=**0** at time *T*. Note that the latter inequality in (1.7) implies that, for convex curves,

$$L^{2}\leq CA^{2\pi /\beta }+4\pi A,$$

so if a solution exists up to extinction, the length must vanish; this eliminates the possibility of extinction as a line of finite length, for example. We define the extinction shape γ* by rescaling *γ* with respect to its arc length *L*, and taking *t* to the extinction time:

2.9 $$\gamma^{*}=\lim_{t\to T}\left\{\left.\frac{\text{x}}{L(\gamma (t))}\;\right|\text{x}\in \gamma (t)\right\}.$$

By this definition *L*(γ*)=1. Of course, one can rescale with respect to other lengths which will give the same extinction shape up to a scaling constant, a fact we shall use in the numerical computations in §2c.

#### (ii) Circles

Consider a curve *γ* represented in polar coordinates by x=r(u,t)(cos⁡u,sin⁡u), for *u*∈[0,2*π*). If *γ*(0) is a circle (i.e. *r*(*s*,0)=*R*(0) is constant) then *γ* remains a circle over time, with a radius *R*(*t*) that satisfies

2.10 $$R\frac{dR}{dt}=-\frac{\beta }{2\pi },\;R(t)=\sqrt{R{\left(0\right)}^{2}-\frac{\beta t}{\pi }}.$$

However, it is of interest as to how generic circular extinction is: whether it is universal for any simple initial condition *γ*(0), or for a large class of initial conditions, or if it only occurs when *γ*(0) is a circle exactly.

To provide some illumination on this question, we perform a linear asymptotic analysis of a near-circular curve. Assume the ansatz

2.11 $$r(u,t)=R(t)+\epsilon R{\left(t\right)}^{a_{n}}\cos(nu)+O(\epsilon^{2}),\;\epsilon \ll 1,\;n\geq 2.$$

The first non-trivial mode of perturbation is *n*=2, as *n*=0 corresponds to a change in spatial scale, while *n*=1 corresponds (up to order *ϵ*) to a spatial translation, under which (1.2) is invariant. If the exponent *a*_*n*_>1, then the asymptotic series remains formally valid (i.e. the correction term does not become larger than the leading order term) as R→0, and the extinction shape γ* is a circle from the definition (2.9). Substituting (2.10) into (1.2), (1.4) and keeping terms to O(ϵ), we find

2.12 $$a_{n}=\frac{2\pi (n^{2}-1)}{\beta }.$$

For standard curve shortening flow (1.1), where *β*=2*π*, we have *a*_*n*_=*n*^2^−1>1 for all *n*≥2.

When *β*>2*π*(*n*^2^−1), then *a*_*n*_<1; an initially small *n*th mode perturbation will grow in comparison to the leading order radius, and we cannot expect the extinction shape of a non-circular initial condition to be a circle. This suggests the existence of other extinction shapes. The smallest value of *β* for which this occurs is when *n*=2 and *β*=6*π*.

#### (iii) Slits

The fact that the second mode *n*=2 grows the fastest when *β*>6*π* suggests a possible extinction shape is the *slit* $\gamma^{*}=[-\frac{1}{4},\frac{1}{4}]$ (note that the length *L*(γ*) is unity, as the interval is covered twice). We denote this behaviour as *slit extinction*.

To establish the existence of such an extinction shape, suppose *γ*(0) is a thin rectangle (with rounded ends, although this is not important in the following) centred about the origin, with *x*-intercepts *x*=*l*(*t*) and *y*-intercepts *y*∼*ϵl*^*b*+1^ as l→0, with *ϵ*≪1. If *b*>0, the *y*-intercept goes to zero faster than the *x*-intercept, and the boundary *γ* approaches the slit shape at extinction. The area and length are *A*∼4*ϵl*^*b*+2^ and *L*∼4*l*, respectively. As the curvature is negligible except near the intercepts, (1.2), (1.4) gives

2.13 $$\frac{d}{dt}(\epsilon l^{b+1})=\frac{2\pi -\beta }{L}.$$

From here we can find d*l*/d*t* and thus express *b* in terms of *β*:

2.14 $$\frac{dl}{dt}=-\frac{\pi }{2\epsilon l^{b+1}},\;b=-2+\frac{\beta }{2\pi }.$$

The slit extinction shape is therefore possible when *β*>4*π*. Given the result (2.12) for a circle, there is an interval *β*∈(4*π*,6*π*), where both circle and slit extinction is possible.

A more detailed asymptotic analysis of the behaviour of thin bubbles near the slit extinction shape, particularly matching to the rounded ends of the bubble where curvature is large, is included in [11].

#### (iv) *n*-fold symmetric extinction shapes

The flow rule (1.2) is an example of an isotropic flow rule: that is, one in which the normal velocity *V* =*V* (*k*) does not explicitly depend on *θ*. For any isotropic flow, if *γ* has initial condition *γ*(0) with *n*-fold symmetry, this symmetry will be preserved over time. For convex curves, this may be demonstrated using the evolution of curvature parametrized by angle (2.4), while if convexity is not assumed we must use the arclength-parametrized equation (2.3). We demonstrate the latter approach here.

In terms of the curvature *k*(*s*,*t*), *n*-fold symmetry corresponds to periodicity with the period an integer fraction of arclength *L*. Say *γ*(*t*) is *n*-fold symmetric at a time *t*:

$$k(s,t)=k\left(s+\frac{L(t)}{n,t}\right).$$

For isotropic flows, this periodicity also implies *V* (*s*,*t*)=*V* (*s*+*L*(*t*)/*n*,*t*). Now we show that the rate of change of the difference of the two curvatures vanishes:

$$\begin{array}{rl} \frac{\partial }{\partial t}\left[k\left(s+\frac{L(t)}{n,t}\right)-k(s,t)\right] & ={\left[\frac{\partial k}{\partial t}\right]}_{s}^{s+L/n}+\frac{1}{n}{\left.\frac{\partial k}{\partial s}\right|}_{s+L/n}\frac{dL}{dt}\\ & ={\left[\frac{\partial^{2}V}{\partial s^{2}}+k^{2}V\right]}_{s}^{s+L/n}+{\left[v\frac{\partial k}{\partial s}\right]}_{s}^{s+L/n}+\frac{1}{n}{\left.\frac{\partial k}{\partial s}\right|}_{s+L/n}\frac{dL}{dt}\\ & =\frac{1}{n}{\left.\frac{\partial k}{\partial s}\right|}_{s}\left({\left[nv\right]}_{s}^{s+L/n}-\frac{dL}{dt}\right)=\frac{1}{n}{\left.\frac{\partial k}{\partial s}\right|}_{s}\left({\left[v\right]}_{0}^{L}-\frac{dL}{dt}\right)=0. \end{array}$$

The last two equalities follow from the periodicity of ∂*v*/∂*s*=*kV* and the last of (1.3).

Given the above argument, if *γ*(0) has *n*-fold symmetry with *n*>2, then the slit extinction behaviour described above in §2b(iii) is not possible. The extinction shape must be either the circle or some other shape with the required symmetry. From the linear stability result (2.12), it is clear that a circle is unstable to *n*-fold perturbations when

2.15 $$\beta >2\pi (n^{2}-1).$$

Thus, for sufficiently large *β*, we expect the existence of further extinction shapes where *n* satisfies (2.15). We compute these extinction shapes numerically in the next section; some typical shapes are depicted in figure 2.

Each of these *n*-fold symmetric extinction shapes is associated with a self-similar solution of the form

2.16 $$k(\theta ,t)=\frac{h(\theta )}{{\left(T-t\right)}^{1/2}},$$

where the length of *γ* is given by *L*(*t*)=*L*(0)(1−*t*/*T*)^1/2^ and the isoperimetric ratio *L*^2^/4*πA* is constant for all time. The function *h* satisfies the second-order ordinary differential equation:

$$\frac{d^{2}h}{d\theta^{2}}+h-\frac{1}{2h}+\mu =0,\;\text{where}\;\mu ={\left(\frac{4\pi A}{L^{2}}\right)}^{1/2}\left(\frac{\beta -2\pi }{2\sqrt{\pi \beta }}\right),$$

which can be integrated once to give

$${\left(\frac{dh}{d\theta }\right)}^{2}=\ln h-h^{2}+1-2\mu (h-1).$$

Here, without loss of generality, we have set d*h*/d*θ*=0 at *h*=1, which could be forced to hold at *θ*=0. The task of computing a periodic *h* from here requires numerical computation. We do not pursue this approach as our more general numerical scheme outlined below in §2c(i) can compute these self-similar solutions easily enough. Again, we note that the extinction shapes in figure 2 also represent the self-similar solutions discussed here.

### (c) Numerical results on extinction behaviour

#### (i) Numerical scheme

To corroborate and expand upon the formal results above, we numerically compute the evolution of curves according to (1.2), (1.4) and extinction shapes for various *β*. There are a variety of approaches we could use for this purpose, many of which are related to the level set method [32–34]. However, for our purposes it is sufficient to employ a rescaled spectral collocation scheme which is a simplification of that we used in [11,35] to solve the problem with an external Laplacian field.

First, for a characteristic length scale λ(*t*) (not necessarily, the length *L*(*γ*)), we define a spatial variable ***X***=λ^−1^***x*** and time scale τ=−log⁡λ. Thus as extinction is approached, λ→0 and τ→∞, while the rescaled curve *γ* tends to a fixed shape which is a scaled version of the extinction shape γ* defined in (2.9). Performing the rescaling, and taking the dot product with the normal ***n***, (1.2), (1.4) becomes

2.17 $$\left(\text{X}-\frac{\partial \text{X}}{\partial \tau }\right)\cdot \text{n}=B(\tau )(K(\tau )-Q(\tau )),\;B=\frac{1}{\lambda \;d\lambda /dt},\;K=\lambda k,\;Q=\lambda q.$$

Only the normal component is required to specify the evolution of the curve, as any tangent velocity amounts to a reparametrization of the curve. Now the boundary is defined in the complex plane (X↦Z∈C) as the image of the unit circle |*ζ*|=1 under the time-dependent power series transformation

2.18 $$Z=G(\zeta ,\tau )=c_{-1}\zeta^{-1}+\sum_{m=1}^{\infty }c_{m}(\tau )\zeta^{nm-1}.$$

Here *n*-fold symmetry is assumed, with the centre of the curve at the origin; taking one line of symmetry to be the real line, each of the *c*_*m*_ are real, which simplifies the numerical procedure.

The rescaled equation (2.17) becomes

2.19 $${\left|\frac{\zeta \partial G}{\partial \zeta }\right|}^{-1}ℜ\left\{\left(G-\frac{\partial G}{\partial \tau }\right)\bar{\zeta \frac{\partial G}{\partial \zeta }}\right\}=B(\tau )\left({\left|\zeta \frac{\partial G}{\partial \zeta }\right|}^{-3}ℜ\left\{\zeta \frac{\partial }{\partial \zeta }\left(\zeta \frac{\partial G}{\partial \zeta }\right)\bar{\zeta \frac{\partial G}{\partial \zeta }}\right\}-Q(\tau )\right).$$

The variable *B* may be determined by enforcing the constant decrease in area, which gives

2.20 $$B(\tau )=\frac{\beta }{2\pi }\left(-c_{-1}+\frac{dc_{-1}}{d\tau }+\sum_{m=1}^{\infty }(nm-1)\left(c_{m}-\frac{dc_{m}}{d\tau }\right)\right).$$

The length scale λ is specified by fixing the leading order coefficient *c*_−1_=1. Now if the series (2.18) is truncated at *N* terms, evaluating (2.19) at *N*+1 evenly spaced points on the arc |*ζ*|=1, arg⁡ζ∈[0,π/n) provides fully implicit equations for the evolution of the unknowns *c*_*m*_(*t*), 1<*m*<*N* and *Q*(*τ*). The evolution of these variables is then computed using the fully implicit solver ode15i in MATLAB. The numerical results presented in this paper were computed using *N*=128.

As well as time evolution of curves, we may compute extinction shapes (which are steady states in the rescaled system) by setting ∂*G*/∂*τ*=0 in (2.19), and solving the resultant algebraic equations using Newton's method. A useful trick in this case is to fix a geometric parameter, such as the aspect ratio for a twofold symmetric shape, and then let the area rate of change *β* be a free parameter that is determined by the Newton scheme. For more general *n*-fold symmetry, the aspect ratio may be generalized by the trough–peak ratio, which we define to be the ratio of minimum to maximum distance from the centre to the curve. By specifying this parameter and solving for *β*, the scheme will converge to the desired non-trivial solution, rather than the circle, which is a solution for any *β*.

#### (ii) Circles and slits

**Figure 1.**
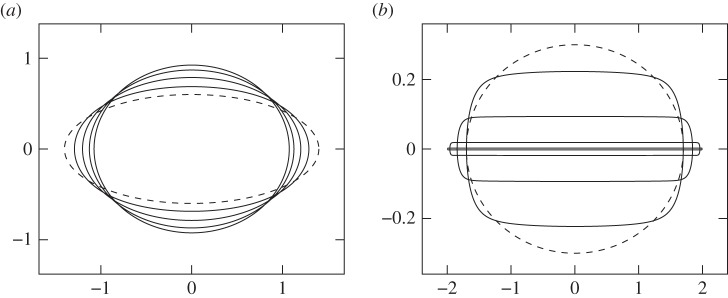
The evolution of a curve computed using the rescaled numerical method of §2c, for *β*=16.9364∈(−6*π*,−4*π*). In (*a*), an initial ellipse (dashed) tends to a circle, and in (*b*), a slightly thinner initial ellipse approaches a slit, demonstrating the two extinction shapes predicted by the asymptotic theory in §2b.

Now we report some results of the numerical scheme. Solving the time-dependent problem assuming twofold symmetry *n*=2, and for *β*∈(4*π*,6*π*), demonstrates that both circle and slit extinction may occur, depending on initial condition. In figure 1, the results of two numerical computations are shown for *β*≈16.94. The initial conditions for the two cases are ellipses with different aspect ratios *α*≈0.43 and *α*≈0.18 (set by choosing *c*_1_(0)=0.4 and 0.7, respectively, and *c*_*m*_(0)=0 for *m*>1). The curve with larger initial aspect ratio tends to a circle as τ→∞, while the one with smaller aspect ratio tends to a slit.

**Figure 2.**
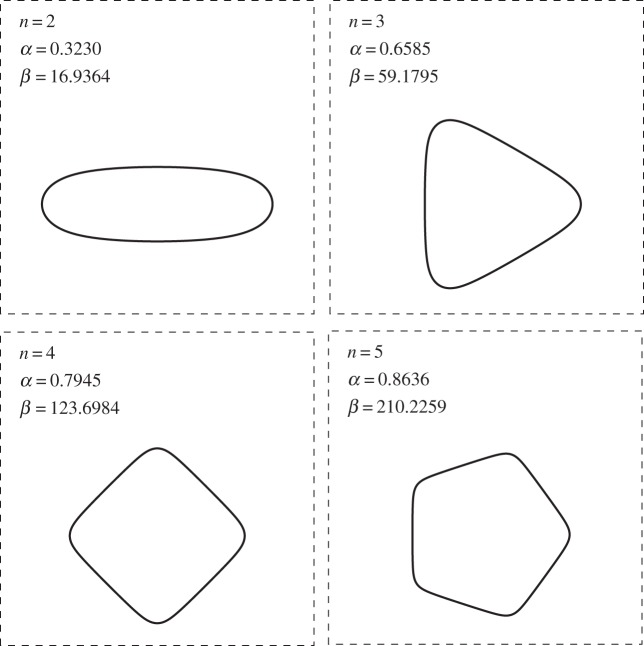
Examples of *n*-fold symmetric extinction shapes computed using the numerical scheme outlined in §2c. Trough–peak ratio *α* and area rate of change *β* are listed for each case. Note these shapes also correspond to self-similar solutions of the form (2.16).

**Figure 3.**
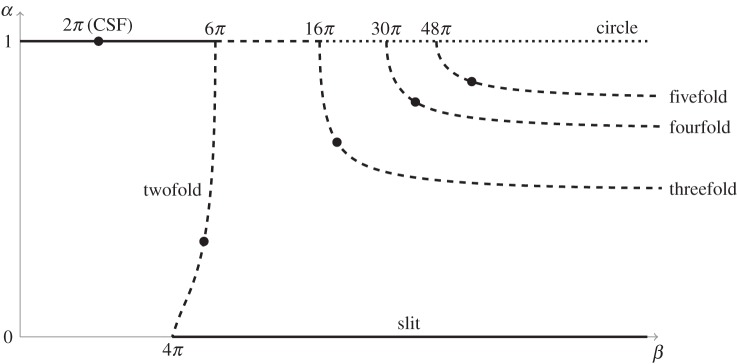
Numerically computed branches of extinction shapes: rate of change of area *β* (plotted logarithmically) against trough–peak ratio *α*. Points marked correspond to extinction shapes plotted in figure 2. Also marked is the point at *β*=2*π*, corresponding to standard curve shortening flow (1.1), where it is known that the circle is the only possible extinction shape.

The existence of these two stable states implies that for *β*∈(4*π*,6*π*), there is also an extinction shape, unstable in rescaled time *τ*, whose aspect ratio lies between one and zero. Indeed, by using the above numerical scheme, we find the existence of such an extinction shape, which is a non-elliptical oval, whose aspect ratio depends on *β*. The extinction shape corresponding to *β*≈16.94 is plotted in figure 2. We also track the dependence of the aspect ratio *α* on the area rate of change *β* (as described above, this is achieved by fixing *α* and determining *β* numerically). This branch of extinction shapes is plotted in figure 3; it connects to the branch of circular extinction shapes as β→6π from below and to the slit extinction shapes as β→4π from above.

#### (iii) *n*-fold symmetry

A similar process as above is used to compute the extinction shapes if *n*-fold symmetry is assumed for *n*>2. In this case, an extinction shape may be characterized by its trough–peak ratio *α*, which as previously described is a generalization of the aspect ratio of a two-fold symmetric shape. Three further examples of extinction shapes are shown in figure 2, with three-, four- and fivefold symmetry enforced in each case. These solutions exist for *β* above the threshold predicted in (2.15).

We also tracked the dependence of *α* on *β*; the resultant branches are plotted in figure 3. While these branches connect to the branch of circular extinction shapes as *β* approaches the threshold value from (2.15), it was found that as β→∞, the extinction shape on each branch tended toward the regular polygon of the given symmetry. Thus, the trough–peak ratio is limited in each case to the value of the polygon, that is

2.21 $$\alpha_{\min}(n)=\cos\left(\frac{\pi }{n}\right),\;n=2,3,\ldots .$$

It is worth emphasizing that the *n*-fold symmetric self-similar solutions for *n*≥3 are likely to be unstable to generic perturbations (but stable to *n*-fold symmetric perturbations). These results are reminiscent of the unstable *n*-fold symmetric contracting bubbles in a Hele-Shaw cell with a power-law type viscous fluid [36,37]. The instability in that model which gives rise to such *n*-fold symmetric shapes is also linked to slit-type extinction shapes in the same way as in this study. Similar phenomena occur in the contracting boundary problem for the porous medium equation [38–41].

### (d) Conjectures

The formal and numerical results above suggest the generalized flow rule (1.2), (1.4) has a much richer range of extinction behaviour than standard curve shortening flow (1.1). It is hoped that these results will motivate the search for rigorous results in the same vein, if focus is restricted to the case of convex initial conditions. Firstly, as we have not found any non-circular extinction shapes for *β*<4*π*, we expect the Gage–Hamilton theorem (which applies only to *β*=2*π*) to extend down to this value:

**Conjecture 2.2**

*An initially convex curve evolving according to* (1.2), (1.4) *with* 0<*β*<4*π* *will approach a round circle in the limit it shrinks to a point, with convergence in the* $C^{\infty }$ *norm*.

More generally, we posit that the extinction shapes discussed in this section cover all possibilities; that is:

**Conjecture 2.3**

*The extinction shape of an initially convex curve evolving according to* (1.2), (1.4) *is one of: circle, slit or* *β*-*dependent* *n*-*fold symmetric shape* (*n*=2,3,…), *depending on initial condition and value of* *β* *as discussed above*.

## 3. Self-intersection

When *q*≠0 in (1.2), (1.4), the possibility arises of an initially simple closed curve intersecting itself before it contracts to a point. This is known to happen in area preserving flow (*β*=0, or *q*=2*π*/*L*) [8], and can lead to curvature singularities in *γ* [10] if the evolution of the non-simple, post-self-intersection curve is considered.

Here we consider curves only up to self-intersection. We present numerical evidence that self-intersection can occur in (1.2), (1.4) for both *q*<0 (*β*<2*π*) and *q*>0 (*β*>2*π*). Self-intersections correspond to a change in topology of the interior of the curve *γ*; given the application to fluid mechanics (for which the closed curve *γ*(*t*) represents the boundary of a shrinking bubble) [11], it is valuable to distinguish between self intersection leading to *pinch-off*, where the interior of *γ* becomes disconnected, and *coalescence*, where the interior of *γ* becomes doubly connected. A rigorous definition may be as follows (recall that ***n*** is an inward-pointing normal):

**Definition 3.1**

Let x*=***x***(*u*_1_)=***x***(*u*_2_), where *u*_1_≠*u*_2_, be a point of intersection. Given a normal boundary velocity *V* (*u*)=∂***x***/∂*t*⋅***n***, order *u*_1_ and *u*_2_ such that |*V* (*u*_1_)|≥|*V* (*u*_2_)|. If *V* (*u*_1_)=−*V* (*u*_2_), then x* is a *degenerate* point of intersection. Otherwise,

— if *V* (*u*_1_)>0, then x* is a *pinch-off* point,
— if *V* (*u*_1_)<0, then x* is a *coalescent* point.

**Figure 4.**
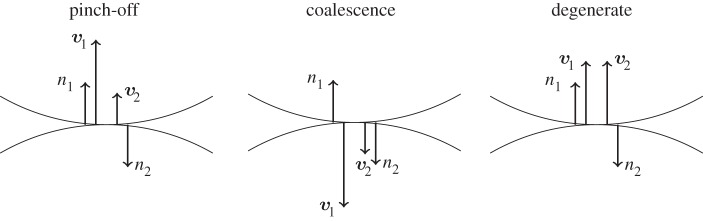
Three different points of self-intersection. Pinch-off occurs when the larger velocity ***v***_1_=*V*_1_***n***_1_ points inward, coalescence when ***v***_1_ points outward, and the point is degenerate if both velocities have the same magnitude and direction (hence opposite signs, as the normals ***n*** point inward).

The three cases are depicted in figure 4.

Intuitively, the term *q* in (1.2) forces a curve *γ* to contract faster if *q*<0 (*β*>2*π*) and more slowly if *q*>0 (*β*<2*π*), than curvature on its own would effect. For *q*<0, therefore, we expect the possibility of pinch-off points, while *q*>0 allows for coalescent points. We again develop a numerical scheme to test these predictions. Unlike the analysis of extinction behaviour, where time and space rescaling is important, here the evolution of an initial curve only needs to be tracked for a short amount of time, so a more common front tracking method is used. A boundary is discretized by a finite number of points ***x***_*n*_, and the normal and curvature are computed using centred finite differences. The velocity of the points is then given directly from (1.2), (1.4).

**Figure 5.**
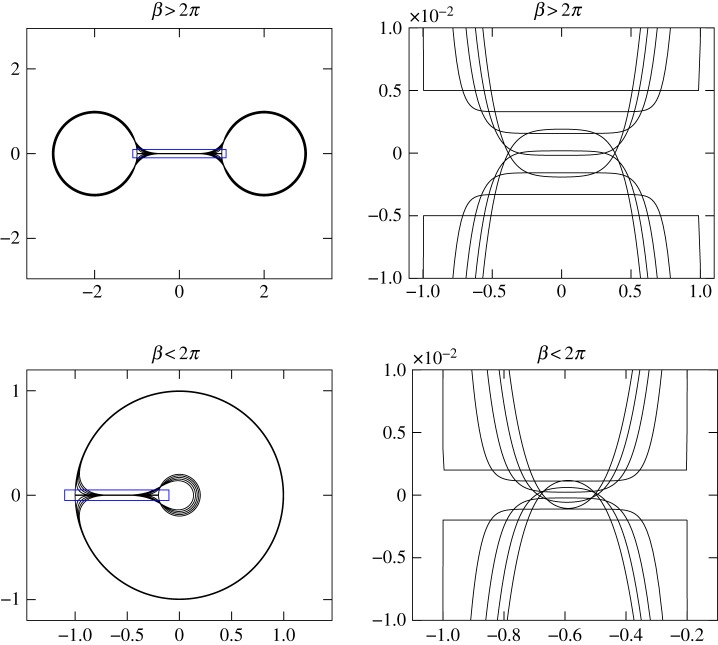
Numerical demonstration of pinch-off (for *β*>2*π*) and coalescence (for *β*<2*π*) due to generalized curve shortening flow (1.2), (1.4). The right-hand panels show the evolution of the curves near the point where self-intersection occurs. (Online version in colour.)

In figure 5 we plot solutions for different values of *β*, such that (a) *q*<0 and (b) *q*>0. The initial condition *γ*(0) is a dumbell and horseshoe shape, respectively. In (a), *q* is small enough to overcome the effect of curvature and close the neck of the dumbell. In (b), *q* is sufficiently large to push the ends of the horseshoe together, again overcoming curvature. Thus, the most important aspect of standard curve shortening flow (1.1), that is, that initially embedded curves remain embedded over time, is not preserved for either *q*<0 or *q*>0.

While the numerical solutions in figure 5 (and others not presented here) have demonstrated that self-intersection is clearly a possibility for non-convex curves, it is likely we can make a statement on the kind of self-intersection that may occur, depending on the sign of *q*: in particular, that *q*<0 allows for pinch-off points, and *q*>0 allows for coalescent points. Both regimes may include degenerate points: for instance, given the right initial condition the ends of the horseshoe or edges of the dumbell neck in figure 5 may just touch and then move apart, or may remain touching for a finite time interval.

To be precise we form the following conjecture. Clearly, any curve shortening problem may have a curve with pinch-off or coalescent points as its initial condition. We must therefore consider the evolution of a boundary according to (1.2), (1.4) over a finite time interval:

**Conjecture 3.2**

*Suppose a curve* *γ*(*t*), *evolving according to generalized curve shortening flow* (1.2), (1.4), *is simply connected in the time interval* (*t*_0_,*t*_1_), *and that* *γ*(*t*_1_) *is not a single point. Then*:

— *if* *β*=2*π* (*CSF*), *then* *γ*(*t*_1_) *is simply connected (no self-intersection*),
— *if* *β*>2*π* (*q*<0), *then* *γ*(*t*_1_) *does not have any coalescent points*,
— *if* *β*<2*π* (*q*>0), *then* *γ*(*t*_1_) *does not have any pinch-off points*.

## 4. Discussion

We have considered here an interesting flow rule (1.2), (1.4) for embedded curves which is notable for the rich array of possible extinction shapes and self-similar solutions as well as the possible modes of self-intersection. The special case *β*=2*π* gives rise to the standard curve shortening flow for which non-convex become convex in finite time, and also any convex curve remains convex until curves contract to a round point. For our generalized curve shortening flow rule with *β*>0, convexity is also preserved, but the subsequent extinction shapes range from circles to slits to *n*-fold symmetric shapes (which appear like regular polygons with rounded corners). On the other hand, embeddedness is not necessarily preserved for (1.2), (1.4) with *β*≠2*π*, leading to the possibility of self-intersection via pinch-off or coalescence.

As mentioned in the introduction, we have employed formal asymptotics and numerical schemes to attack this problem, with the hope that our conjectures could be studied using more rigorous analysis. In addition, there are further questions that deserve attention. For example, for the range 4*π*<*β*<6*π*, in which it appears that both circular and slit extinction is possible, it would presumably be a challenge to derive a criterion that distinguishes between initial convex curves that evolve to either one or the other. Similarly, regarding the possibilities of self-intersection for initially non-convex curves, a difficult problem would be to determine which initial curves remain embedded and which do not.

## Data accessibility

This work does not have any experimental data.
